# Trends and determinants of mortality in women of reproductive age in rural Guinea-Bissau, West Africa – a cohort study

**DOI:** 10.1186/1472-6874-13-48

**Published:** 2013-12-05

**Authors:** Maram Mane, Ane B Fisker, Henrik Ravn, Peter Aaby, Amabelia Rodrigues

**Affiliations:** 1 Projecto de Saúde de Bandim, INDEPTH Network, Bissau, Guinea-Bissau; 2 Escola Nacional de Saúde, Instituto Nacional de Saúde Pública, Bissau, Guinea-Bissau; 3 Research Center for Vitamins and Vaccines (CVIVA), Statens Serum Institut, Copenhagen, Denmark

**Keywords:** Women of reproductive age, Mortality, West Africa

## Abstract

**Background:**

There are few studies reporting mortality of women of reproductive age (WRA) in developing countries. The trend and patterns of their mortality may be important for documenting the health status of the population in general.

**Methods:**

We used a prospective open cohort of women aged 12 to 49 years living in the Bandim Health Project’s rural Health and Demographic Surveillance System (HDSS) in 5 regions of Guinea-Bissau from 1996 to 2007. Information on in- and out-migration and deaths were collected through the HDSS routine procedures. We assessed the trends in mortality and the associated determinants using Cox regression models.

**Results:**

We followed 27,185 WRA for 141,693 person-years-at-risk (PYO) among whom 9,093 moved out and 1,006 died. Overall standardized mortality rate was 759 per 100,000 PYO. WRA mortality did not decline, but three periods could be distinguished: a stable mortality between 1996–2000 followed by 14% increase in mortality [Hazard rate ratio (HRR) = 1.14; 95% confidence interval (CI): 0.98-1.32; p = 0.08] between 2001–2003, and then in the last period from 2004–2007 a 25% decline (HRR = 0.75; 95% CI: 0.64-0.87; p < 0.001) in relation to the first period. Compared with the years 1990–1996 mortality increased in the first two periods until 2003; only in the last period did mortality reach the same level as in 1990–1996 (HRR = 0.96; 95% CI: 0.82-1.13; p = 0.62). The level of mortality differed between regions. In the adjusted analysis the eastern regions Bafata (HRR = 1.79; 95% CI: 1.38-2.32; p < 0.001) and Gabu (HRR = 1.70; 95% CI: 1.28-2.26; p < 0.001) had significantly higher mortality, but the hazard rate did not differ by ethnic group. As expected the rate increased with increasing age.

**Conclusions:**

Over the twelve-year period mortality of WRA did not decline. A stable mortality in the beginning was followed by an increase and then a return to the previous levels. Further monitoring of mortality is needed to identify the risk factors for the striking regional differences. Advantage should be taken of the HDSS to monitor progress towards the MDGs and beyond.

## Background

Despite calls for more attention to adult health in low-income countries, little change has been seen in terms of availability of reliable data and in terms of policy interventions [1-3]. The health policies focus mainly on childhood interventions due to high risk of child mortality and the assumption that after surviving the childhood period, the risk of adult death is low; however, this belief has been challenged in developing countries since the risk of adult death is still considerable and is apparently not declining in sub-Saharan Africa [3,4]. In community studies in Africa, adult mortality rate of around 730 per 100,000 person-years of observation (PYO) was seen in Burkina Faso for the period of 1993–1998 and in Tanzania for a period of 2003–2007 [5,6]. An analysis conducted by Rajaratnam and colleagues of empirical data from different sources in several countries showed great variability of adult mortality between countries and over time [3]. Unfortunately, little reliable data exists on adult mortality in Africa due to lack of official registration of deaths [7,8] and the data available comes from sources of varying reliability [3,9]. Data on causes of deaths is even worse since many deaths occur in the community and establishing the causes would require gathering information through interviews with close relatives [8,10]. Our ignorance on the levels of mortality and causes of deaths may also have contributed to the reluctance to design interventions targeting adult health in sub-Saharan Africa.

Even though some adult health issues like HIV/AIDS, tuberculosis, and maternal mortality are targeted by international initiatives, it would be difficult to establish their burden or the impact of the interventions without information on the total adult mortality [6,9]. Maternal mortality is the fifth millennium development goal (MDG5) but progress in reaching the goal has been slow in sub-Saharan Africa, with no progress in certain countries [11]. Maternal mortality is influenced by general health risks faced by WRA; thus, progress in reducing maternal mortality would certainly imply capacity to identify and act on other causes of WRA mortality [12].

The importance of WRA in African society is indisputable. Apart from the biological function of bearing the future human beings, they are the basis for childcare and education, household production and wellbeing of the family, care of older people, and an important economic force in the countries [12]. The death of a woman will impact negatively on the child, even though the extended family culture usually absorb orphans and mitigate the impact; for young children death of the mother leads to a much higher mortality [13,14].

There are few community studies of adult female mortality from Africa apart from those related to maternal mortality [3-5]. Available community data showed high mortality rates: 720, 780 and 1410 per 100,000 PYO, respectively, in Tanzania between 2003–2007, in Ethiopia between 1987–2004, and in Burkina Faso between 1993–1998 [6,15,16]. Maternal mortality may represent one-third of all deaths of WRA [17], but HIV/AIDS contributes also substantially to the high mortality of WRA [18].

Guinea-Bissau has little information on the magnitude and causes of WRA mortality since official registration of deaths is lacking as in many other African countries. The UNICEF Multiple Indicators Cluster Surveys (MICS) organized every 3 years provided in the past only maternal mortality estimates using the sisterhood method [19]. The only data on WRA mortality is from the Health and Demographic Surveillance System (HDSS) of the Bandim Health Project (BHP) from rural districts between 1990 and 1996. The WRA mortality rate was 581 per 100,000 PYO and 32% of the deaths were related to complications during pregnancy, the maternal mortality ratio being 811 per 100,000 live births [20].

Prediction of adult mortality is a difficult task since the level and trends do not always follow general expectations; for instance adult mortality does not necessarily decline in the presence of declining child mortality, and in some countries the level of mortality does not correspond to the income level [3,21,22]. Hence, we need reliable local data assessing the role of economic development, social wellbeing, health services, social unrests, new diseases, and cultural factors for adult mortality and particularly the mortality of women of reproductive age. Since they are usually the most vulnerable, societal changes may easily influence WRA mortality and their mortality level could be used to select interventions contributing to the health of the general population.

Thus, taking advantage of the existing longitudinal data collection of BHP, we studied the trends and determinants of mortality among women of reproductive age in rural Guinea-Bissau in the period 1996–2007.

## Methods

### Study site and population

The study was conducted in rural areas of Guinea-Bissau, West Africa. The general census conducted in 2009 estimated at total population of around 1.5 million, of whom 51.8% were women and 49.6% of these were of childbearing age; 60% of the population lived in rural areas [23]. Guinea-Bissau is among the poorest countries in the world, ranking 164^th^ in terms of health; life expectancy is 48.6 years [24]. The administrative structure includes 8 regions and the capital Bissau: Biombo, Cacheu and Oio regions are situated in the North, Bafata and Gabu the in the East, and Tombali, Quinara and Bolama in the South.

The study performed by the BHP HDSS was initiated in 1990 with selection of 20 clusters of villages in each of the five northern and eastern regions; data collection in the southern regions was only initiated in 2006. Women of reproductive age and their children less than five years of age constitute the rural HDSS population. The present study included only the 100 clusters from the northern and eastern regions followed since 1990.

### Design

We used the prospective open cohort of women aged between 12 and 49 years of age living in the rural HDSS villages during the period from 1^st^ January, 1996 to December 31^st^, 2007.

### Registration and follow-up

The BHP registered all WRA and their children in the baseline census. The WRA and their children have been followed longitudinally through six-monthly home visits; the only exception was the war period from May 1998 to May 1999 when visits were not conducted; however, visits were resumed as soon as the war stopped. During each round, all compounds and households are visited and information is updated on all individuals already registered and new eligible women and children are included. All girls of at least 10 years of age and showing signs of puberty (breast, menstruation) are registered as are WRA moving in to live in the HDSS area. A unique identification number (ID) is assigned to each individual. The women’s ID is built from the codes of the region, village, compound and WRA number; the latter number is a running number within the compound and is assigned to a woman when first registered.

At each visit, all new pregnancies are registered and the outcome of previously registered pregnancies is ascertained. Hence, all vital events are registered, such as pregnancies, births, in- and out-migration, and deaths. If the person has out-migrated information is collected on where the person moved to and when. If a death is encountered a short questionnaire is applied to determine the major causes of death. The date of the event is registered as accurately as possible using as reference known events. If it is not possible to know the exact day, usually the month is known, so the 15^th^ of the month is used as the date.

The main background information on the WRA includes the name, name of the relative with whom she is living (which could be the father, husband, aunt or someone else), age, ethnic group, uptake of anti-tetanus toxoid vaccine, ante-natal consultations for pregnant women and where they gave birth. Verbal autopsy interviews were performed to determine the probable causes of death.

### Ethics

The longitudinal registration and follow-up as part of the HDSS was approved by the Ministry of Health in the beginning of the study. All individuals entering the HDSS cohort are asked to consent verbally. The data analysis was approved by the Comité Nacional de Ética em Saúde of Guinea-Bissau as part of a PhD research protocol.

### Data management and analyses

The BHP databases are stored in Dbase V and were transferred to STATA 11 for analysis. Mortality hazard rates (HR) and hazard rate ratios (HRR) were calculated. The outcome was death from any cause occurring within the study period and age group range. The follow-up period started the 1^st^ of January 1996 or when the WRA was first registered and terminated when death occurred, she completed 49 years of age or on the 31^st^ of January 2007. Women moving out of the study area were censored on that date. Since the follow-up lasted for several years, the data was split into time-span records at each year and age group, thus women could contribute to different age group categories over time. Age groups were categorized as 12 to 14, 15 to 19, 20 to 24, 25 to 29, 30 to 34, 35 to 39, 40 to 44, and 45 to 49 years old. Age was estimated from the age at the date of registration. Five main ethnic groups were identified plus one mixed group with all the small ethnic groups. The overall mortality standardized by general regional population was estimated.

We used Cox proportional hazards regression model to estimate hazard ratios and corresponding 95% confidence intervals; a test for trend was done for period and age group. The mortality rate in the current period was compared to the previous reported WRA mortality rate in 1990–96 [20].

## Results

Between 1^st^ January, 1996, and 31^st^ December, 2007, a total of 27,274 women of reproductive age were identified as living in the rural HDSS in the 5 regions. Among them, 89 (0.3%) were excluded for the following reasons: 48 were duplicates, 40 did not have information on age and one had date of entry after the exit date. The dates are only accurate with respect to month since the exact day was known only for 18% of deaths and 9% of migrations. Nearly all could report the month of the event.

The remaining 27,185 WRA were followed for 141,693 person-years-at-risk (PYO). The mean follow-up time was 5.2 years. During the study period, 9,093 moved out of the area and 1,006 died. The median age of WRA at the time of death was 26 years (Interquartile range: 20–35) and the youngest woman was 12 years old. The main probable causes of death determined by verbal autopsy were maternal mortality (33%), HIV/AIDS (16%) and tuberculosis (14%).

**Figure 1:**
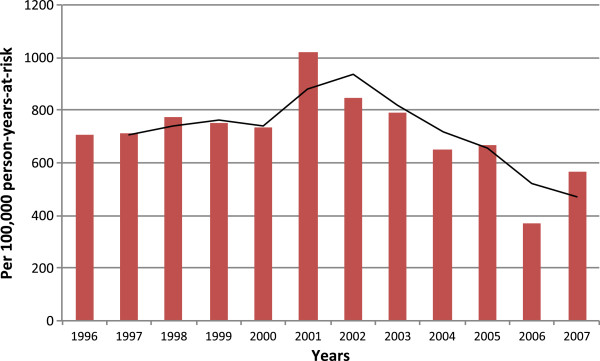
**Overall trend in mortality of women of reproductive age, 1996–2007.** Hazard mortality rate per 100,000 person-years-at-risk over 1996–2007 is presented.

The overall standardized mortality rate was 759 per 100,000 PYO (95% confidence interval [CI]: 743–774). Over the 12 years of follow-up mortality did not decline (adjusted test for trend: p = 0.09). Three periods could be distinguished (Figure 1) – a stable mortality in the beginning followed by an increase and then a decline. In the first 5 years, the mortality rate varied between 707 in 1996 and 775 per 100.000 PYO in 1998 (Table 1); In 2001, there was a sudden increase representing the highest HR for the whole 12 years period (1023 per 100,000 PYO). Mortality then declined slightly, but in this period, from 2001 to 2003, mortality increased 14% (HRR = 1.14; 95% CI: 0.98-1.32; p = 0.08) compared to the previous five years period. Then, from 2004 to 2007, a significant 25% decrease occurred (HRR = 0.75; 95% CI: 0.64-0.87; p < 0.001).

**Table 1:** Mortality rates of women of reproductive age over the period, 1996-2007

**Determinant**	**Deaths**	**Person-years-at-risk**	**Rate per 100,000**	**95% Confidence interval**
**Period**				
1996	75	10606	707	564–887
1997	80	11270	710	570–884
1998	88	11360	775	629–955
1999	85	11318	751	607–929
2000	84	11446	734	593–909
2001	119	11635	1023	855–1224
2002	99	11712	845	694–1029
2003	94	11928	788	644–965
2004	79	12183	648	520–808
2005	81	12138	667	537–830
2006	49	13172	372	281–492
2007	73	12925	565	449–710
**Region**				
**Northern**				
Cacheu	154	28722	536	458–628
Biombo	183	32776	558	483–645
Oio	190	27888	681	591–785
**Eastern**				
Bafata	245	26446	947	836–1074
Gabu	234	25850	885	778–1006
**Ethnic group**				
Fula	297	34467	862	769–966
Mandinga	214	27258	785	687–898
Balanta	207	27573	751	655–860
Pepel	155	27392	566	483–662
Manjaco	64	13037	491	384–627
Other	68	11784	577	455–732
**Age group**				
12–14	4	2123	188	71–502
15–19	109	23958	455	377–549
20–24	137	29435	465	394–550
25–29	154	24753	622	531–729
30–35	163	20886	780	669–910
35–39	141	17941	786	666–927
40–44	106	13891	763	631–923
45–49	192	8708	2205	1914–2540
**Overall standardized mortality**	1006	141693	759	743–774

Mortality rates in these periods were compared to mortality in the 1990–1996 period when the WRA mortality rate was 581 per 100,000 PYO. During the period from 1996 to 2000 mortality was 27% higher (HRR = 1.27; 95% CI: 1.09-1.46; p = 0.001); from 2001 to 2003, mortality continued to increase (HRR = 1.52; 95% CI: 1.30-1.78; p < 0.001); however, from 2004 to 2007 the HR returned to the antecedent level (HRR = 0.96; 95% CI: 0.82-1.13; p = 0.63).

**Figure 2:**
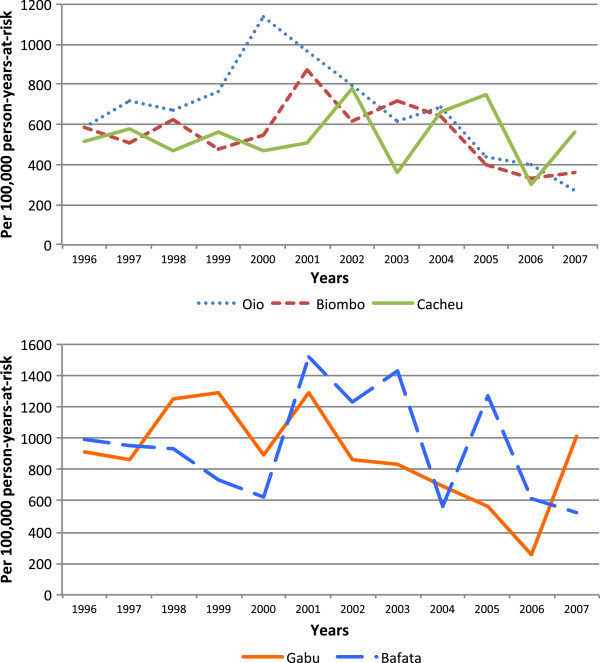
**Trends in mortality of women of reproductive age by region, 1996–2007.** Hazard mortality rates per 100,000 person-years-at-risk by northern (Biombo, Cacheu and Oio) and eastern (Bafata and Gabu) regions are presented.

The trend in mortality differed between regions. In Gabu, mortality was extremely high in 1998, 1999 and 2001 (HR = 1256, 1293 and 1293 per 100,000 PYO respectively) and then a steady decline occurred until an increase to 1010 was observed again in 2007 (Figure 2). In Bafata, the highest mortality was observed between 2001 and 2003 with rates of 1516, 1229 and 1427 per 100,000 PYO, respectively, and with another peak in 2005 (HR = 1270 per 100,000 PYO). In the north - in Oio, Cacheu and Biombo - mortality increased slowly from 1996 (HR = 583 per 100,000 PYO) attaining the peak in 2000 (HR = 1134 per 100000 PYO) in Oio, and then a steady decrease occurred until 2007 (HR = 271 per 100,000 PYO).

In the unadjusted analysis, mortality was higher in the eastern regions, Bafata (HR = 947 per 100,000 PYO) and Gabu (885 per 100,000 PYO), compared with the northern regions where the HR was 536 per 100,000 in Cacheu, 558 per 100,000 PYO in Biombo and 681 per 100,000 PYO in Oio. Compared with Cacheu, Bafata had 74% (p < 0.001) and Gabu 62% (p < 0.001) higher mortality. Among the northern regions, mortality in Oio was 25% (p = 0.03) higher than in Cacheu, but Biombo (p = 0.83) had the same HR as Cacheu (Table 2). The same pattern was observed for the ethnic groups consistent with the region where they are the majority. The ethnic groups Fula and Mandinga, the majority in the eastern regions, and Balanta living in Oio had higher mortality (p < 0.01) compared with Pepel, the main group living in Biombo.

**Table 2:** Determinants of mortality of women of reproductive age, 1996–2007

**Determinant**	**Unadjusted analysis**			**Adjusted analysis**		
	**Hazard ratio**	**95% Confidence interval**	**p-value**	**Hazard ratio**	**95% Confidence interval**	**p-value**
**Period**						
**1996–2007**	0.99	0.99–0.99	<0.001*	0.99	0.99–1.00	0.09*
**Region**						
Cacheu	1	-	-	1	-	-
Biombo	1.02	0.83–1.27	0.83	0.82	0.55–1.21	0.32
Oio	1.25	1.01–1.55	0.03	1.06	0.83–1.37	0.62
Bafata	1.74	1.42–2.13	<0.001	1.79	1.38–2.32	<0.001
Gabu	1.62	1.32–1.99	<0.001	1.70	1.28–2.26	<0.001
**Ethnic group**						
Pepel	1	-	-	1	-	-
Balanta	1.35	1.10–1.66	0.00	1.06	0.74–1.54	0.74
Mandinga	1.39	1.13–1.70	0.00	0.83	0.55–1.26	0.37
Fula	1.53	1.26–1.85	<0.001	0.78	0.51–1.20	0.25
Manjaco	0.88	0.66–1.17	0.37	0.74	0.46–1.18	0.20
Other	1.04	0.78–1.38	0.81	0.72	0.46–1.12	0.14
**Age group**						
12–14	0.44	0.16–1.19	0.11	0.43	0.16–1.17	0.10
15–19	1	–	–	1	–	–
20–24	1.00	0.77–1.30	0.99	0.99	0.76–1.28	0.91
25–29	1.40	1.08–1.80	0.01	1.35	1.04–1.76	0.02
30–34	1.79	1.39–2.30	<0.001	1.70	1.31–2.21	<0.001
35–39	1.80	1.38–2.34	<0.001	1.72	1.30–2.26	<0.001
40–44	1.82	1.37–2.41	<0.001	1.72	1.28–2.32	<0.001
45–49	5.17	4.02–6.66	<0.001	4.88	3.73–6.39	<0.001
			<0.001*			<0.001*

*Test for trend.

Regarding age group, mortality increased with age after 25 years of age compared to young women aged 15–19 years old (test for trend: p < 0.001). After 45 years of age mortality was five-fold higher than in the age group 15 to 19 years old. The age groups from 30 to 44 years had similar mortality levels (Table 2).

In the Cox proportional hazard model adjusting for period, region, age group, and ethnic group, the main predictors of WRA mortality were living in the eastern regions and increasing age after 25 years of age. Mortality was at least 70% higher in Bafata (HRR = 1.79; 95% CI: 1.38-2.32; p < 0.001) and in Gabu (HRR = 1.70; 95% CI: 1.28-2.26; p < 0.001). Women aged 25 to 29 years had a 35% higher mortality than women aged 15–19 years old (HRR = 1.35; 95% CI: 1.04-1.76; p = 0.02) and mortality increased with increasing age category (test for trend: p < 0.001). The age categories from 30 to 44 presented 70% higher mortality (p < 0.001) and the last age category 45 to 49 years of age had almost a five-fold excess mortality (HRR = 4.88 95% CI: 3.73-6.39; p < 0.001).

## Discussion

The twelve years of longitudinal follow-up in rural Guinea-Bissau showed no decline in mortality of women of reproductive age. Although fluctuations and regional differences were noticed, three periods could be distinguished. Between 1996–2000, mortality increased 27% compared with the previous period (1990–96) [20]. Two major events may have influenced mortality levels in this period.

First, a civil war occurred from June 1998 to May 1999, with fighting mainly around Bissau city. Numerous internally displaced people were present in the surroundings of Bissau, especially in Biombo [25,26], but many fled as far as Bafata, Gabu and Oio. Almost all returned to their home during relatively long cease-fire periods. The mortality levels in the present study are for residents in the rural areas who were not directly exposed to the war. However, general disruption of social and economic activities, stress in food availability, and intensified disease transmission due to relatives fleeing from Bissau could have contributed to overall increased mortality. The cultural rules for hospitality obliging residents to receive displaced people at home and to share food with them and the policy of humanitarian aid agencies to provide relief-food only to non-residents could have placed the residents in a vulnerable situation [26]. This may have been the situation in Biombo where almost all villages received displaced people and a slight increase in mortality was seen in 1998. Gabu also had increased mortality during the war in 1998–1999. In the regions of Bafata, Gabu and Oio internally displaced people were mainly concentrated in the towns where non-governmental organizations such as Médicin Sans Frontière and PLAN Guinea Bissau (Bafata), Catholic organization (Oio and Biombo) and branches of the United Nations Agencies as WHO, UNICEF, and WFP (Bafata) operated.

The second major event was a meningitis epidemic in the whole country from January to May 1999 with 2860 cases and 483 deaths reported to the epidemiological surveillance service of the Ministry of Health. The most affected regions were Oio with 1150 cases and 76 deaths, Cacheu with 543 cases and 160 deaths, Bafata with 483 cases and 69 deaths, and Gabu with 367 cases and 76 deaths. Only 6% of the deaths occurred among individuals over 15 years old. The meningitis epidemic could only have contributed to the remarkable mortality increase in Gabu since few deaths were registered in Biombo and in the other regions mortality increased only in later years.

Between 2001 and 2003 all regions experienced at least one peak in mortality. This period was characterized by political, military and economic instability, several threats and at least one coup d’état in 2003. There was a further increase in mortality to 884 per 100,000 PYO in this period. Intensified transmission of HIV-1 during and after the war, facilitated by the presence of foreign troops [27] and the concentration of national troops in strategic regions may have contributed to this trend. HIV prevalence among women in Bissau doubled from 1997 to 1999, but then stabilized [27,28]. HIV infection may have developed rapidly to AIDS during this period. In the cohorts followed in Bissau city, HIV-infected individuals had marked excess mortality during the war period compared with HIV-uninfected individuals suggesting that HIV-infected were particularly affected by the reduced access to health care and drugs (authors’ unpublished data). The post-war stress period was not mitigated by antiretroviral treatment, which only became available in 2005 [29].

A 25% decrease in HR was observed in 2004–2007 in relation to the period 1996–2000; nonetheless, there were increases in mortality in Bafata in 2005 and in Gabu in 2007. Mortality was still at the level of 1990–1996. Thus, at the moment, it is not possible to confirm that mortality is declining in rural Guinea-Bissau; only continued monitoring can clarify whether mortality is declining.

Data on mortality of women of reproductive age is nearly non-existent for Sub-Saharan Africa. For the available studies methodologies differ making comparisons difficult. Thus, mainly mortality rates from other HDSS are available. The mortality was 720 per 100,000 PYO in Ifakara HDSS in Tanzania from 2003–2007. Although their analysis included older women, whose mortality would increase the overall rate, the level does not seem very different from Guinea-Bissau [6]. Some misclassification of age group may also have been present in our data since older rural women usually do not know their exact age and some older women might have been classified in the 45–49 years age group. An increase in mortality was reported in Ifakara between 2003 and 2007. Both the Ifakara study and our data sadly confirm no decline in adult female mortality in rural sub-Saharan Africa as also reported by others [2,6,30,31].

The HIV epidemic is thought to be the cause of the increased mortality in adults [2,31]. In Guinea-Bissau, the regions of Gabu and Bafata have the highest prevalence of HIV among pregnant women and Gabu also had the highest mortality in the previous study [32]. In the national HIV sentinel survey performed in 2009, the prevalence of HIV infection was 10.3% in Bafata and 9.6% in Gabu among pregnant women, but in the northern regions it was only around 3.5% [Fronteira *et al*., unpublished observations]. Indeed, Bafata (HRR = 1.79; p < 0.001) and Gabu (HRR = 1.70; p < 0.001) had significantly higher WRA mortality corroborating the above hypothesis. We did not find ethnic group to be associated with mortality in the adjusted Cox model; however, it is difficult to disassociate ethnicity from region.

Data on adult female mortality in sub-Saharan Africa is scarce. Complete vital registration in Africa is lacking and will probably not be available soon [8]. The current study contributes with direct estimates of mortality rates from community data. Furthermore, the longitudinal follow-up allows us to depict changes over time and importantly to identify regional differences. Unfortunately few variables were included in the analysis since the HDSS routine data collection is limited; however, the regional and ethnic determinants may aggregate the effect of other determinants such as cultural and behavioural characteristics, biological/genetic susceptibility, exposure to biological, environmental conditions, and access to health services among others.

HDSS sites may not be representative of a whole country and may have a different health status due to interventions carried out there. However, the rural HDSS in Guinea-Bissau consist of randomly selected clusters of villages in each region and there was no specific adult intervention implemented in these villages suggesting that the sample may well be representative for the rural areas. One influence could be that asking about pregnancy and providing free pregnancy cards could prompt women to go to antenatal consultations. The value of HDSS data lies in its longitudinal character which allows tracking changes over time in a more precise way since reporting of deaths is more complete than in cross-sectional surveys.

## Conclusions

Over the twelve-year period mortality of WRA did not decline. The tendency for decrease observed in the last period needs confirmation over the next years. While we are counting down to the Millennium Development Goals [18], accurate data for their evaluation are needed. It is important to pursue monitoring WRA and adult mortality in general and to identify the determinants of mortality. It will be important in Guinea-Bissau to re-appraise the priorities from a broader perspective and to have an in-depth understanding of the causes of the striking regional differences in order to design specific interventions for the eastern regions. HDSS can contribute greatly to the knowledge of mortality trends and patterns in adults and specifically of WRA.

## Abbreviations

BHP: Bandim health project; CI: Confidence interval; CVIVA: Research Center for Vitamins and Vaccines; HDSS: Health and demographic surveillance system; HIV: Human immunodeficiency virus; AIDS: Acquired immunodeficiency syndrome; HR: Hazard rate; HRR: Hazard rate ratio; ID: Identification number; INDEPTH: International network for demographic evaluation of populations and their health in developing countries; MDG: Millennium development goal; MICS: Multiple indicator cluster survey; PYO: Person years of observation; UNICEF: United Nation Children’s Fund; VA: Verbal autopsy; WRA: Women of reproductive age; WFP: World Food Programme; WHO: World Health Organization.

## Competing interests

The authors declare that they have no competing interests.

## Authors’ contributions

MM, AR, and PA planned the study. PA is the leader of the BHP HDSS and AF, HR and AR contributed to the implementation of the HDSS. MM and AR coordinated the verbal autopsies data collection and classification. AR and HR conducted the analyses. MM wrote the first draft and all authors contributed to the final version. All authors read and approved the final manuscript.

## Pre-publication history

The pre-publication history for this paper can be accessed here:

http://www.biomedcentral.com/1472-6874/13/48/prepub
